# Case report: A 53-year-old woman with synchronous WHO classification II and IV gliomas

**DOI:** 10.3389/fonc.2024.1308497

**Published:** 2024-06-11

**Authors:** Fang Jia, Yin Kang, Zhanxiang Wang

**Affiliations:** Department of Neurosurgery, Xiamen Key Laboratory of Brain Center, the First Affiliated Hospital of Xiamen University, School of Medicine, Xiamen University, Xiamen, China

**Keywords:** glioblastoma, astrocytoma, IDH, WHO grade, synchronous, case report

## Abstract

**Introduction:**

Glioma is the most common primary intracranial neoplasm with a relatively poor prognosis.

**Case presentation:**

Here, we present a unique case of a 53-year-old woman with two histopathologically distinct gliomas at the initial diagnosis. She presented with headaches and left limb weakness before admission, and magnetic resonance imaging (MRI) showed right frontal and basal ganglia area involvement combined with hemorrhage. The patient underwent a navigation-guided craniotomy for tumor removal. Pathological examination revealed the right frontal lobe lesion as a WHO grade II IDH-NOS astrocytoma, but the right parietal lobe lesion was a WHO grade IV IDH-mutant diffuse astrocytoma. Molecular detection of the parietal lesion revealed a point mutation at the R132 locus of the *IDH1* gene, no mutation in the *TERT* promoter, amplification of the epidermal growth factor receptor, and a non-homozygous *CDKN2A/B* deletion.

**Discussion:**

In-depth epigenomic analysis and molecular examination revealed that one patient had two different brain tumors, underscoring the importance of performing a comprehensive brain tumor workup.

**Conclusion:**

This unique case confirms that adjacent astrocytomas may have different molecular pathogenesis and provides novel insights into the development of gliomas.

## Introduction

In 2021, the World Health Organization (WHO) released the fifth edition of the Classification of Tumors of the Central Nervous System (1). In this updated edition, IDH-mutant glioblastoma, formerly known as “secondary glioblastoma”, is now described as IDH-mutant, grade IV astrocytoma. Additionally, astrocytoma with IDH-mutant grade III and synonymous *CDKN2B* and/or *CDKN2A* deletions were also characterized as astrocytoma, IDH mutations, grade IV, albeit with a relatively low histopathologic grade (1, 2). The detailed interaction mechanism between IDH1/2 and CDKN2A/B remains unclear at the biomolecular level. Current research has focused more on the discrepancies between grade 4, IDH-mutant astrocytoma, and glioblastoma (3–6). Few clinical studies have been conducted specifically on this phenotype, and current knowledge remains limited (5).

The simultaneous presence of multiple foci, remarkably homologous foci with different histopathologic compositions, is rare in all types of gliomas (7). Based on radiologic and/or pathologic features, complicating lesions can be divided into multifocal and multicentric categories (8). Multicentric gliomas fail to differentiate due to genetic defects in stem cells, resulting in defective, highly proliferative cell populations that form tumor centers (9). Multifocal gliomas, on the other hand, represent the presence of multiple tumor foci that are differentially associated and have consistent genetic variants (8). Due to this rarity, the underlying molecular relationships of synchronous lesions are poorly understood. Thus far, despite the worse prognosis of multifocal/multicentric gliomas, their treatment is roughly the same as for single lesions (9).

The co-occurrence of astrocytoma of different molecular and histologic classifications at initial diagnosis in a single individual has never been previously described in the literature. We emphasized the importance of integrated genetic and pathology analysis for synchronous gliomas.

## Case presentation

**Figure 1:**
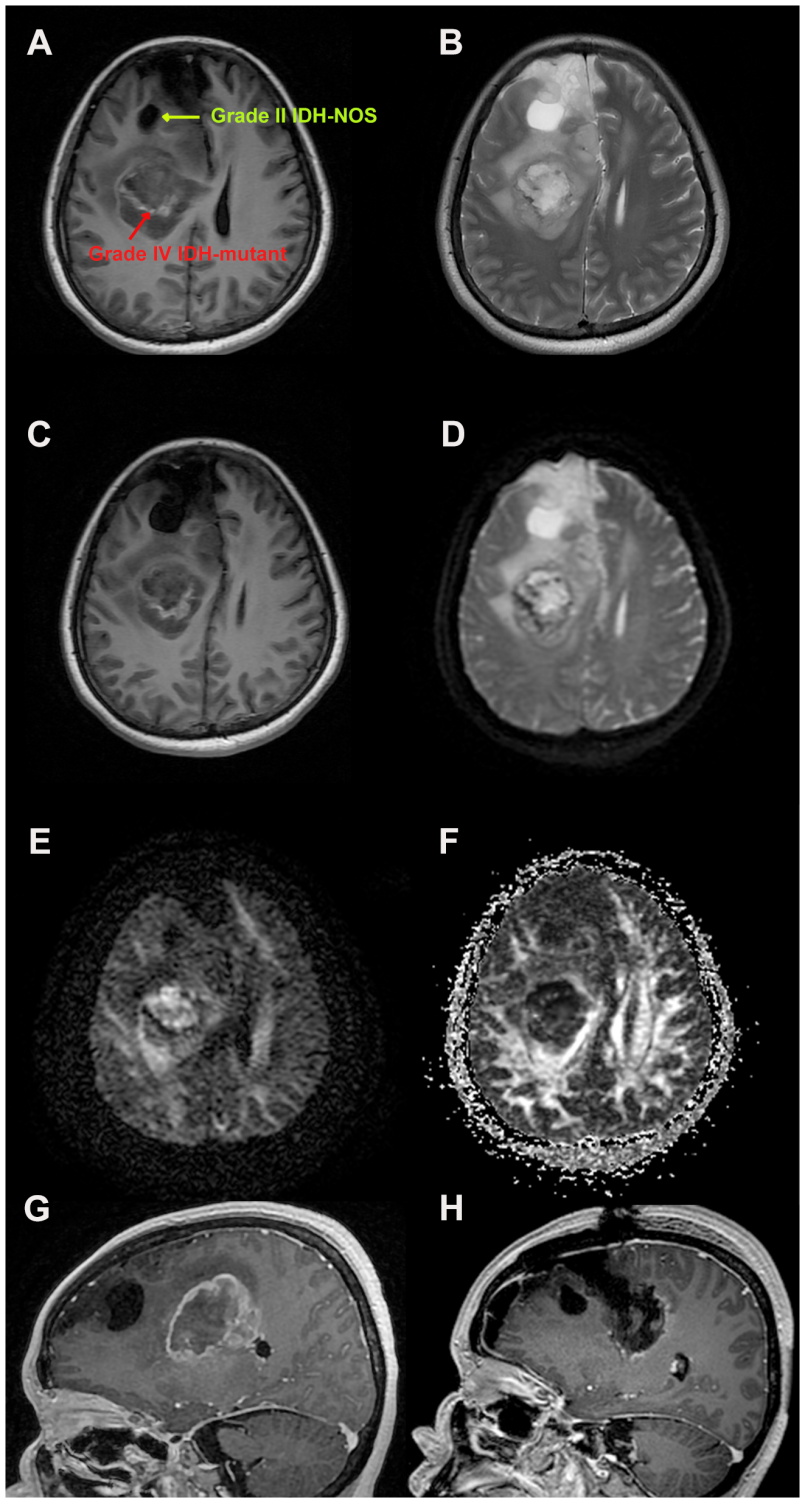
Magnetic resonance imaging findings during the patients’ treatment. **(A)** Axial post-enhancement T1 weighted images showing a right-sided contrast lesion in the parietal and basal ganglia area, measuring 5.1*4.2*3.7 cm^3^, and a right frontal lobe cystic mass. **(B)** Axial post-enhancement T2 weighted Propeller images to avoid artifacts. **(C)** Heterogenous, T1 weighted isometric lesion in the basal ganglia area, with midline shift and sub-calcaneal hernia. **(D)** Axial T1-FLAIR-weighted images showing parietal lesions as heterogeneous lesions with high contrast frontal cystic masses. Diffusion tensor imaging **(E)** showing the cerebral neurofibril trajectory and factional anisotropy **(F)**. **(G)** Preoperative sagittal T1-weighted images reveal two heterogeneous lesions with peripheral edema zone. **(H)** Postoperative sagittal T1-weighted images showing sub-total tumor resection with partial contrast (to avoid neurological deficits).

The patient, a previously healthy 53-year-old woman, attended a local clinic with a progressive headache accompanied by nonprojectile vomiting. Later, the patient’s symptoms worsened, and she developed weakness in the left limb. She underwent an MRI scan, which showed occupational hemorrhage in the right frontal lobe and basal ganglia region and blood accumulation in the knee of the corpus callosum with subcerebral falciform herniation. The larger lesion was located in the right parietal lobe, showing signs of isometric T1-weighted images (T1WIs) and T2WIs and contrast enhancement with peri-lesion edema and midline shift, size approximately 5.1*4.2*3.7 cm^3^ (**Figures 1A, B**). The other lesion was a saccular mass located in the right frontal lobe (**Figures 1C**). Several nodular, clustered bands of edema with hyperintensity on fluid-attenuated inversion recovery (FLAIR) images and diffusion-weighted imaging (DWI) were seen (**Figures 1D–G**). She subsequently underwent a navigation-guided trans-frontal craniotomy using electrophysiology to detect functional brain areas. A sub-total resection (STR) was performed to avoid causing dysfunction in the basal ganglia region. Intraoperative ultrasound was used to assess tumor resection. Postoperatively, the patient’s left-sided muscle strength recovered from grade II to grade IV, and MRI revealed minimal contrast enhancement of residual lesions (**Figure 1H**).

**Figure 2:**
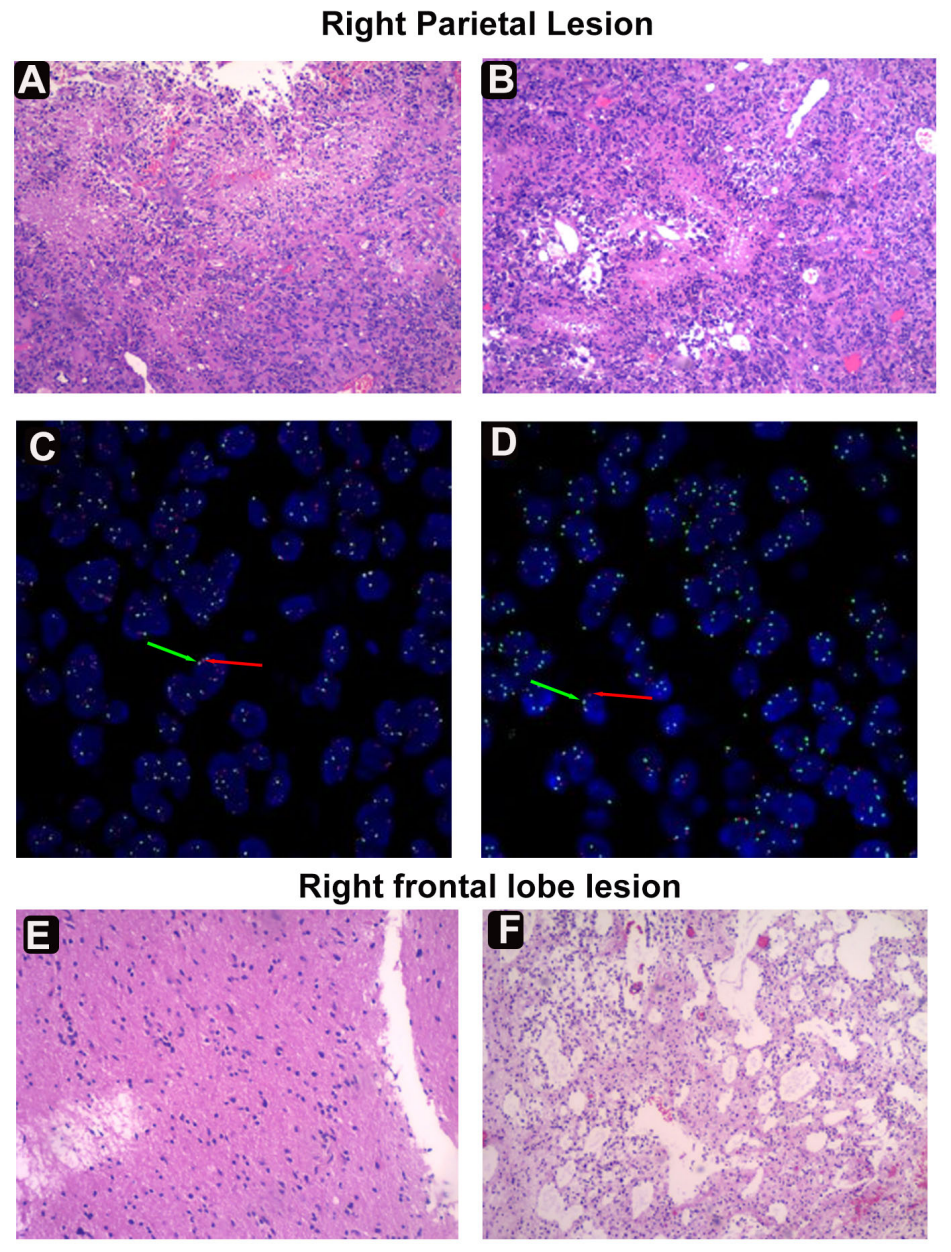
Histological and fluorescence *in situ* hybridization (FISH) examinations. **(A)** Diffuse infiltrating growth of tumor cells seen microscopically. **(B)** Tumor cells of variable size, with coarse chromatin, nucleoli, and nuclear fission. Dense growth of tumorigenic astrocyte-like cells in the Virchow-Robin interstitial. **(C)** FISH assay revealing the EGFR amplification. The red arrow represents the gene EGFR, a total of 207; The green arrow denotes the GSP-17 chromosome, a total of 75. **(D)** FISH assay suggesting *CDKN2A/B* nonpure deletion. Green arrow (G) for/CSP9, red arrow (R) for CDKN2A. **(E)** Tumor cells are infiltrated in sheets of nests, cells are ovoid/short pike with mild cell morphology. **(F)** Lower cell density and microcyst formation in the frontal area.

Histopathological analysis of the parietal lesion demonstrated a WHO grade IV IDH-mutant diffuse astrocytoma with mesenchymal vascular, endothelial cell proliferation, and palisading necrosis. Microscopically, the cells were dense, tightly arranged, and of variable size, coarse chromatin, visible nucleoli, and nuclear schizophrenia was atypical (**Figures 2A, B**). The cells were positive for *GFAP*, *IDH1*, *Olig-2*, *NeuN*, *Nestin*, *NF*, *S100*, *CD34*, *SYN*, *EMN*, and Vimentin, but negative for ATRX by immunohistochemistry. The Ki-67 proliferation index was significantly increased, labeling up to approximately 55% of tumor cells. Most cells showed strong *p53* staining (80%), and immunofluorescence of *EGFR* was positive. Detection of *CDKN2A/B* by fluorescence *in situ* hybridization (FISH) suggested non-purifying deletions (**Figures 2C, D**). PCRR-Fluorescent with QIAamp FFPE Tissue Kit confirmed the presence of *IDH1* R132 mutation and revealed no *TERT* promoter mutations (**Table 1**, **Supplementary Figure S1**). Pathological diagnosis of the frontal lesion was a WHO grade II IDH-NOS (Isocitrate Dehydrogenase-Not otherwise specified) diffuse astrocytoma without necrosis and microvascular proliferation (**Figures 2E, F**). The Ki-67 proliferation index was slightly increased, marking approximately 2% of tumor cells. The cells were positive for p53, and ATRX staining was not observed (**Supplementary Figure S2**). The patient tolerated the operation well, continued to improve clinically, and achieved a Karnofsky Performance Scale (KPS) score of 80 at discharge. The patient will then be treated one month later with concurrent temozolomide (TMZ) and radiation, the standard Stupp regimen. Based on follow-up MRI, the patient is currently in a tolerable disease state (**Supplementary Figure S3**).

**Table 1:** Comparison of the immunohistochemistry and genetic findings between the right frontal and parietal lobe lesions.

Parietal lobe lesion	Frontal lobe lesion
WHO-grade IV, IDH 1 R132 mutation	WHO-grade II, IDH-NOS
Ki67 (55%+), p53 (80% +)	Ki67 (2%+), p53 (+)
TERT-promoter, wildtype	TERT-promoter, wildtype
ATRX (-) by IHC	ATRX (-) by IHC
EGFR-positive, CDKN2A-negative by FISH	

## Discussion

In this study, we describe the first case of a patient with two adjacent but histologically distinct primary gliomas at initial treatment. The most notable finding was a WHO grade IV diffuse astrocytoma with the IDH1 R132 mutation in the right parietal basal ganglia region pathology, which differed from the WHO grade II astrocytoma, IDH-NOS found in the right frontal lobe lesion.

This discovery raises two possible pathogenic mechanisms. One speculation is that the right parietal lesion is a secondary lesion derived from the frontal tumor. The different IDH mutations may suggest an evolved trajectory resulting in more invasive clones able to metastasize transmission (8, 10). In this case, glioma cells in the parietal lesion probably possess a more remarkable ability to metastasize through cerebrospinal fluid or cortical tract fibers. Another speculation is the existence of two separate and synchronously evolving multicentric tumors. Higher proportions of IDH1 mutations have been reported in low-grade astrocytomas compared with those in primary glioblastomas and oligodendrogliomas (11–13). Considering this may be an early clonal mutation, different IDH1 mutations may indicate the simultaneous progression of two separate low-grade gliomas (14). In 1996, Watanabe et al. reported the presence of distinct genetic alterations in what were previously called primary and secondary glioblastomas (15). EGFR alterations are prevalent in primary glioblastoma but infrequent in secondary glioblastoma. P53 mutations are much more common in secondary glioblastoma but rare in primary glioblastoma. Interestingly, both p53 (80% positive) and EGFR alterations were detected in the right parietal lesion. But we do not suggest that these two lesions are bi-primary or completely separate tumors since they share the same P53 and ATRX mutations and are anatomically adjacent. The discoveries suggest that ATRX and P53 mutations may first appeared in glial precursor cells. Afterwards, these cells acquired EGRF mutations and became grade IV diffuse astrocytoma. The remaining cells turned into IDH-NOS grade II astrocytoma. This case suggests that IDH mutations could subsequently occur after other genetic changes. In 2020, Yoon et al. made a similar point (16). In their case, the new lesion was diagnosed as glioblastoma six years after a surgical of IDH-mutant diffuse astrocytoma. In recent years, exosomes have been found to perform an important role in intercellular communication, and exosomes secreted by cancer cells have a powerful ability to alter the distant and local microenvironment (17). We speculate that glioma cells in the frontal and parietal lesions may communicate via nanoscale vesicles in neurons and glial cells.

A literature search identified only two published cases of oligodendrogliomas WHO grade-2 and astrocytomas WHO grade-2 (18). Singha et al. described two similar patients with seizure who suffered from oligodendrogliomas of the left parietal lobe and astrocytomas of the right frontal lobe (18). At 6-month follow-up, the patients’ imaging and clinical status remained stable. However, they only performed routine pathological examinations and immunohistochemical tests and did not provide molecular profiling to demonstrate the different biological backgrounds of the two gliomas reported.

Another critical point is the impact of the novel WHO classification in 2021 on clinical care compared to the WHO classification in 2016 (1, 19). Before this update, the patient’s right parietal tumor was classified as an IDH-mutant glioblastoma and, therefore, should receive a gross tumor resection and adjuvant chemotherapy and radiotherapy (20). Based on the latest 2021 WHO classification, the tumor is currently classified as a WHO grade IV IDH-mutant diffuse astrocytoma. Considering her health condition, we performed a sub-total tumor resection.

## Conclusion

We report here a rare case of adjacent multifocal diffuse astrocytoma with distinct WHO grades, IDH mutations, and biological background. A combination of molecular and histologic parameters uncovered distinct clonal origins of the two lesions. We also highlighted the differences between the 2016 WHO classification and the updated 2021 version regarding glioma classifications.

## Data availability statement

The original contributions presented in the study are included in the article/Supplementary Material. Further inquiries can be directed to the corresponding author.

## Ethics statement

The participants provided their written informed consent for the publication of their potentially-identifying data and/or images.

## Author contributions

FJ: Writing – original draft, Writing – review & editing. YK: Conceptualization, Investigation, Software, Writing – review & editing. ZW: Data curation, Methodology, Supervision, Writing – original draft.

## Supplementary material

Supplementary Figure 1 Detection of TERT, IDH1/2 mutations in tumor individualized therapy-related genes in the parietal lesion.

Supplementary Figure 2 The whole diagnostic histopathology report for this patient.

Supplementary Figure 3 Magnetic resonance images two months after discharge. **(A)** Axial post-enhancement FLAIR images. **(B, C)**. Axial T2-weighted propeller images after enhancement to avoid artifacts. **(D)**. Axial T1-FLAIR weighted images. Diffusion tensor images revealing nerve fiber trajectories **(E)** and anisotropy **(F)** in the brain. Sagittal **(G)** and coronal **(H)** T1-weighted images.
