# Supplementary figures and images for: Case report: A 53-year-old woman with synchronous WHO classification II and IV gliomas
Source: Front Oncol. 2024 Jun 11;14:1308497. doi: 10.3389/fonc.2024.1308497 (PMC11196406; doi:10.3389/fonc.2024.1308497)

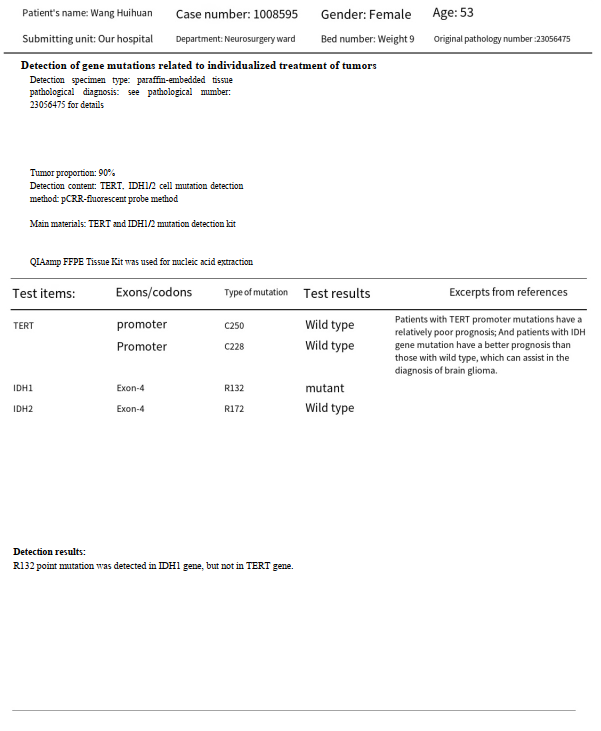

Supplement: Supplementary Figure 1 — Detection of TERT, IDH1/2 mutations in tumor individualized therapy-related genes in the parietal lesion. [file Image_1.tif]

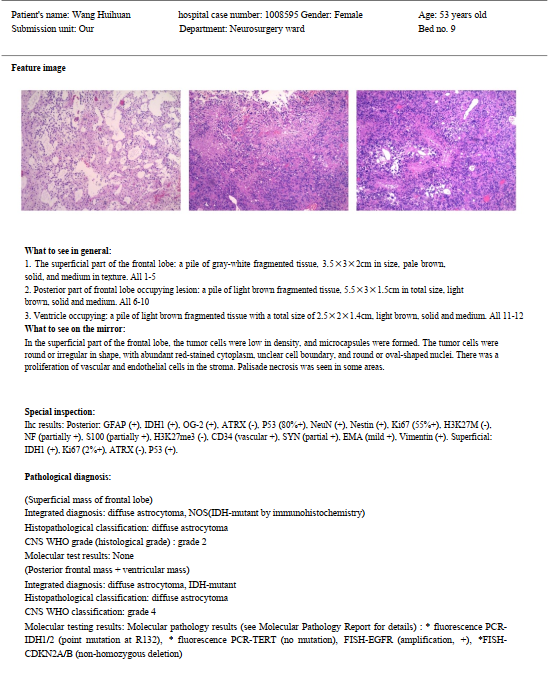

Supplement: Supplementary Figure 2 — The whole diagnostic histopathology report for this patient. [file Image_2.tif]

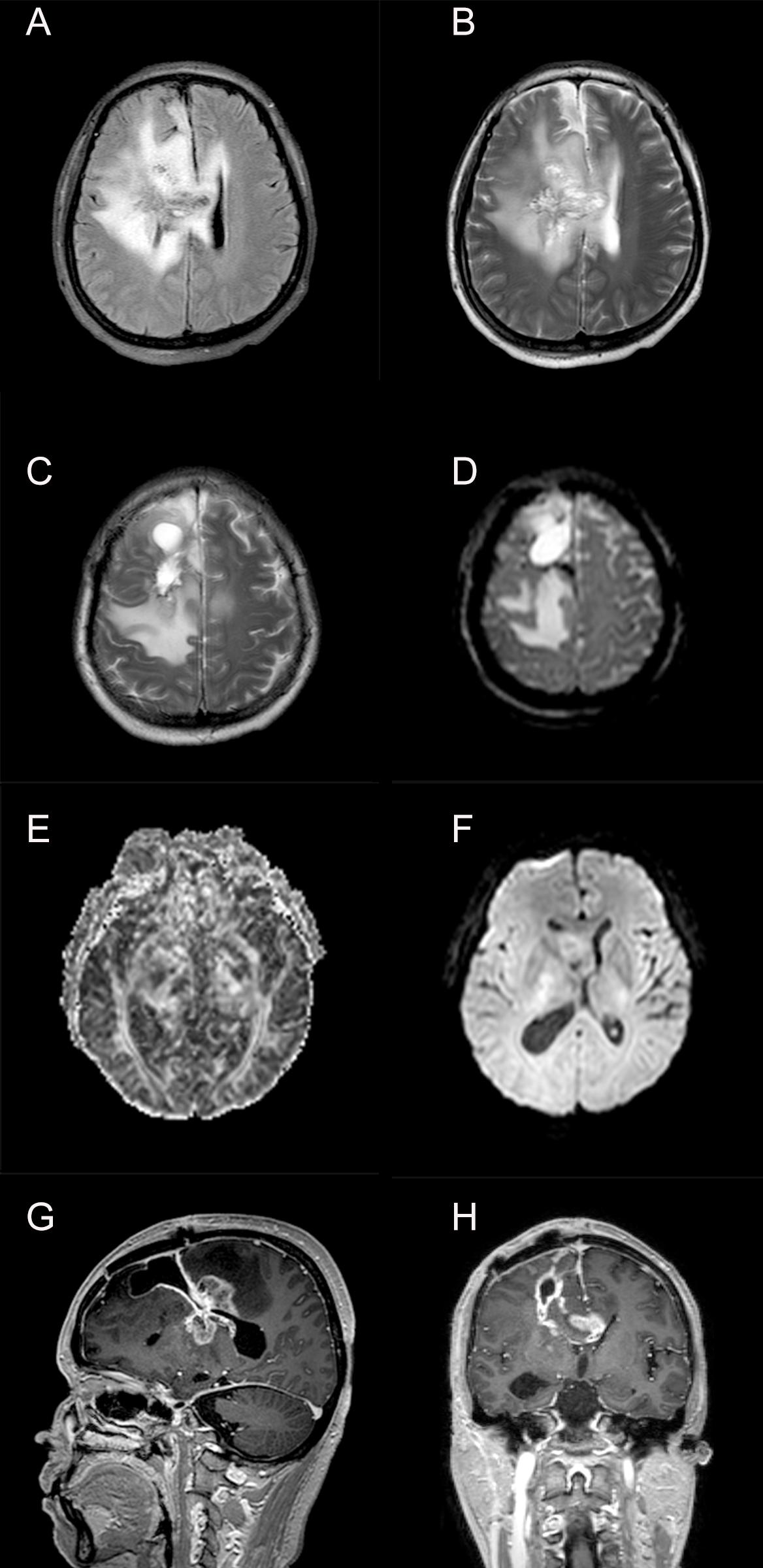

Supplement: Supplementary Figure 3 — Magnetic resonance images two months after discharge. (A) Axial post-enhancement FLAIR images. (B, C). Axial T2-weighted propeller images after enhancement to avoid artifacts. (D). Axial T1-FLAIR weighted images. Diffusion tensor images revealing nerve fiber trajectories (E) and anisotropy (F) in the brain. Sagittal (G) and coronal (H) T1-weighted images. [file Image_3.tif]
